# {4,4′-Dimethyl-2,2′-[2,2-dimethyl­propane-1,3-diylbis(nitrilo­methanylyl­idene)]diphenolato}copper(II) monohydrate

**DOI:** 10.1107/S1600536812034502

**Published:** 2012-08-11

**Authors:** Hadi Kargar, Reza Kia, Fatemeh Ganji, Valiollah Mirkhani

**Affiliations:** a Department of Chemistry, Payame Noor University, PO Box 19395-3697 Tehran, I. R. of IRAN; b Department of Chemistry, Science and Research Branch, Islamic Azad University, Tehran, Iran; c Department of Chemistry, University of Isfahan, 81746-73441 Isfahan, Iran

## Abstract

The asymmetric unit of the title compound, [Cu(C_21_H_24_N_2_O_2_)]·H_2_O, comprises half of a Schiff base complex and half of a water mol­ecule. The whole compound is generated by crystallographic twofold rotation symmetry. The geometry around the Cu^II^ atom, located on a twofold axis, is distorted square-planar, which is supported by the N_2_O_2_ donor atoms of the coordinating Schiff base ligand. The dihedral angle between the symmetry-related benzene rings is 47.5 (4)°. In the crystal, the water mol­ecule that is hydrogen bonded to the coordinated O atoms links the mol­ecules *via* O—H⋯O inter­actions into chains parallel to [001]. The crystal structure is further stabilized by C—H⋯π inter­actions, and by π–π inter­actions involving inversion-related chelate rings [centroid–centroid distance = 3.480 (4) Å].

## Related literature

For applications of Schiff bases in coordination chemistry, see: Granovski *et al.* (1993 ▶); Blower (1998 ▶). For related structures, see: Ghaemi *et al.* (2011 ▶); Kargar *et al.* (2011 ▶, 2012 ▶). For standard bond lengths, see: Allen *et al.* (1987 ▶).
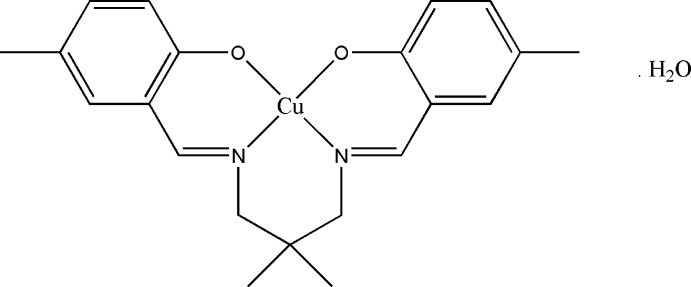


## Experimental

### 

#### Crystal data

[Cu(C_21_H_24_N_2_O_2_)]·H_2_O
*M* *_r_* = 417.98
Monoclinic, 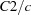

*a* = 13.353 (5) Å
*b* = 15.986 (5) Å
*c* = 10.023 (5) Å
β = 104.696 (5)°
*V* = 2069.5 (14) Å^3^
*Z* = 4
Mo *K*α radiation
μ = 1.08 mm^−1^
*T* = 296 K
0.11 × 0.08 × 0.05 mm

#### Data collection

Bruker SMART APEXII CCD area-detector diffractometer
Absorption correction: multi-scan (*SADABS*; Bruker, 2005 ▶) *T*_min_ = 0.891, *T*_max_ = 0.948
4967 measured reflections
1779 independent reflections
1053 reflections with *I* > 2σ(*I*)
*R*_int_ = 0.101

#### Refinement

*R*[*F* ^2^ > 2σ(*F* ^2^)] = 0.084
*wR*(*F* ^2^) = 0.209
*S* = 0.95
1779 reflections
125 parameters
H-atom parameters constrained
Δρ_max_ = 1.03 e Å^−3^
Δρ_min_ = −0.96 e Å^−3^

### 

Data collection: *APEX2* (Bruker, 2005 ▶); cell refinement: *SAINT* (Bruker, 2005 ▶); data reduction: *SAINT*; program(s) used to solve structure: *SHELXS97* (Sheldrick, 2008 ▶); program(s) used to refine structure: *SHELXL97* (Sheldrick, 2008 ▶); molecular graphics: *SHELXTL* (Sheldrick, 2008 ▶); software used to prepare material for publication: *SHELXTL* and *PLATON* (Spek, 2009 ▶).

## Supplementary Material

Crystal structure: contains datablock(s) global, I. DOI: 10.1107/S1600536812034502/su2488sup1.cif

Structure factors: contains datablock(s) I. DOI: 10.1107/S1600536812034502/su2488Isup2.hkl

Additional supplementary materials:  crystallographic information; 3D view; checkCIF report

## Figures and Tables

**Table 1:** Hydrogen-bond geometry (Å, °) *Cg*1 is the centroid of the C1–C6 ring.

*D*—H⋯*A*	*D*—H	H⋯*A*	*D*⋯*A*	*D*—H⋯*A*
O1*W*—H1*W*1⋯O1	0.85	2.46	2.783 (7)	103
O1*W*—H1*W*1⋯O1^i^	0.85	2.44	2.783 (7)	105
C3—H3⋯O1*W* ^ii^	0.93	2.55	3.48 (1)	173
C8—H8*B*⋯*Cg*1^iii^	0.97	2.83	3.693 (9)	148
C11—H11*B*⋯*Cg*1^iv^	0.96	2.98	3.850 (12)	151

Symmetry codes: (i) 
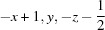
; (ii) 
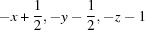
; (iii) 
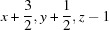
; (iv) 
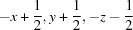
.

**Fig. 1.**
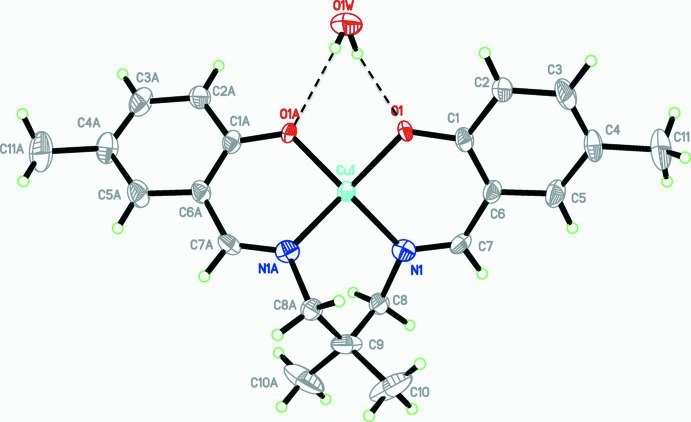
A view of the molecular structure of the title compound, with the atom numbering. The displacement ellipsoids are drawn at the 40% probability level. The O-H···O hydrogen bonds are shown as dashed lines (see Table 1 for details; symmetry code for suffix A = -x+1, y, -z-1/2).

**Fig. 2.**
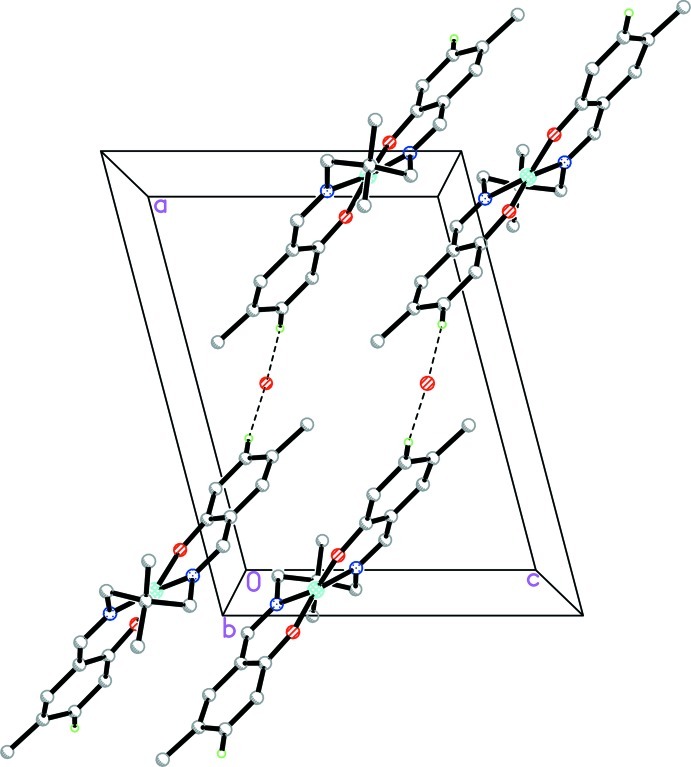
A view along the b axis of the crystal packing of the title compound showing the C—H···O interactions as dashed lines [see Table 1 for details; only the H atoms involved in these interactions are shown].

## supplementary crystallographic information

### Comment

Schiff base complexes are one of the most important stereochemical models in transition metal coordination chemistry, with the ease of preparation and structural variations (Granovski *et al.*, 1993; Blower, 1998). In continuation of our work on the structural analysis of Schiff base metal complexes (Kargar *et al.*, 2012; Kargar *et al.*, 2011; Ghaemi, *et al.*, (2011), we synthesized the title compound and report herein on its crystal structure.

The asymmetric unit of the title compound, Fig. 1, comprises half of a Schiff base complex and half a water molecule. The Cu1 and C9 atoms of the complex and the O atom of the water molecule lie on a two-fold rotation axis which generates the whole complex. The bond lengths (Allen *et al.*, 1987) and angles are within the normal ranges and are comparable to those reported for related structures (Kargar *et al.*, 2012; Kargar *et al.*, 2011; Ghaemi *et al.*, (2011). The geometry around the Cu^II^ atom is distorted square-planar which is supported by the N_2_O_2_ donor atoms of the coordinated Schiff base ligand. The dihedral angle between the substituted benzene rings is 47.5 (4)°.

In the crystal, the water molecule that is hydrogen bonded to the coordinated O atoms, O1, mediates linking of molecules by C—H···O interactions (Table 1 and Fig. 2). The crystal structure is further stabilized by C-H···π interactions (Table 1), and by π-π interactions involving inversion related chelate rings [*Cg*···*Cg*^i^ = 3.480 (4) Å; Cg is the centroid of the Cu1/O1/C1/C6/C7/N1 ring; symmetry code: (i) 1 - *x*, -*y*, -1 - *z*].

### Experimental

The title compound was synthesized by adding 5-methyl-salicylaldehyde-2,2-dimethyl-1,3-propanediamine (2 mmol) to a solution of CuCl_2_. 4H_2_O (2.1 mmol) in ethanol (30 ml). The mixture was refluxed with stirring for 30 min. The resultant solution was filtered. Dark-green single crystals of the title compound suitable for *X*-ray structure determination were recrystallized from ethanol by slow evaporation of the solvents at room temperature over several days.

### Refinement

The water H atom was located in a difference Fourier map and refined as a riding atom with U_iso_(H) = 1.5U_eq_(O). The C-bound H-atoms were included in calculated positions and treated as riding atoms: C—H = 0.93, 0.96 and 0.97 Å for CH, CH_3_ and CH_2_ H-atoms, respectively, with U_iso_ (H) = k x U_eq_(C), where k = 1.5 for CH_3_ H-atoms, and = 1.2 for other H-atoms.

### Crystal data

**Table:**

[Cu(C_21_H_24_N_2_O_2_)]·H_2_O	*F*(000) = 876
*M**_r_* = 417.98	*D*_x_ = 1.342 Mg m^−^^3^
Monoclinic, *C*2/*c*	Mo *K*α radiation, λ = 0.71073 Å
Hall symbol: -C 2yc	Cell parameters from 512 reflections
*a* = 13.353 (5) Å	θ = 2.5–27.4°
*b* = 15.986 (5) Å	µ = 1.08 mm^−^^1^
*c* = 10.023 (5) Å	*T* = 296 K
β = 104.696 (5)°	Block, dark-green
*V* = 2069.5 (14) Å^3^	0.11 × 0.08 × 0.05 mm
*Z* = 4	

### Data collection

**Table:**

Bruker SMART APEXII CCD area-detector diffractometer	1779 independent reflections
Radiation source: fine-focus sealed tube	1053 reflections with *I* > 2σ(*I*)
Graphite monochromator	*R*_int_ = 0.101
φ and ω scans	θ_max_ = 25.0°, θ_min_ = 2.0°
Absorption correction: multi-scan (*SADABS*; Bruker, 2005)	*h* = −15→11
*T*_min_ = 0.891, *T*_max_ = 0.948	*k* = −18→18
4967 measured reflections	*l* = −10→11

### Refinement

**Table:**

Refinement on *F*^2^	Primary atom site location: structure-invariant direct methods
Least-squares matrix: full	Secondary atom site location: difference Fourier map
*R*[*F*^2^ > 2σ(*F*^2^)] = 0.084	Hydrogen site location: inferred from neighbouring sites
*wR*(*F*^2^) = 0.209	H-atom parameters constrained
*S* = 0.95	*w* = 1/[σ^2^(*F*_o_^2^) + (0.0775*P*)^2^] where *P* = (*F*_o_^2^ + 2*F*_c_^2^)/3
1779 reflections	(Δ/σ)_max_ < 0.001
125 parameters	Δρ_max_ = 1.03 e Å^−^^3^
0 restraints	Δρ_min_ = −0.96 e Å^−^^3^

### Special details

**Table:**

Geometry. All esds (except the esd in the dihedral angle between two l.s. planes) are estimated using the full covariance matrix. The cell esds are taken into account individually in the estimation of esds in distances, angles and torsion angles; correlations between esds in cell parameters are only used when they are defined by crystal symmetry. An approximate (isotropic) treatment of cell esds is used for estimating esds involving l.s. planes.
Refinement. Refinement of F^2^ against ALL reflections. The weighted R-factor wR and goodness of fit S are based on F^2^, conventional R-factors R are based on F, with F set to zero for negative F^2^. The threshold expression of F^2^ > 2sigma(F^2^) is used only for calculating R-factors(gt) etc. and is not relevant to the choice of reflections for refinement. R-factors based on F^2^ are statistically about twice as large as those based on F, and R- factors based on ALL data will be even larger.

### Fractional atomic coordinates and isotropic or equivalent isotropic displacement parameters (Å^2^)

**Table:**

	*x*	*y*	*z*	*U*_iso_*/*U*_eq_	
Cu1	0.5000	0.01026 (6)	−0.2500	0.0266 (5)	
O1	0.4089 (4)	−0.0737 (2)	−0.3499 (6)	0.0312 (14)	
N1	0.4554 (5)	0.0944 (3)	−0.3912 (7)	0.0305 (16)	
C1	0.3331 (6)	−0.0612 (4)	−0.4620 (9)	0.0293 (19)	
C2	0.2617 (6)	−0.1276 (4)	−0.5073 (10)	0.042 (3)	
H2	0.2662	−0.1758	−0.4541	0.050*	
C3	0.1860 (7)	−0.1214 (5)	−0.6291 (11)	0.046 (3)	
H3	0.1375	−0.1642	−0.6530	0.055*	
C4	0.1790 (6)	−0.0526 (5)	−0.7191 (10)	0.045 (2)	
C6	0.3222 (6)	0.0109 (4)	−0.5441 (9)	0.0311 (19)	
C7	0.3854 (6)	0.0845 (4)	−0.5067 (10)	0.032 (2)	
H7	0.3753	0.1282	−0.5699	0.038*	
C8	0.5166 (6)	0.1727 (4)	−0.3691 (9)	0.036 (2)	
H8A	0.4984	0.2056	−0.4531	0.043*	
H8B	0.5894	0.1587	−0.3512	0.043*	
C9	0.5000	0.2260 (6)	−0.2500	0.048 (4)	
C5	0.2449 (6)	0.0134 (5)	−0.6699 (10)	0.042 (2)	
H5	0.2380	0.0621	−0.7224	0.051*	
C11	0.1004 (8)	−0.0471 (7)	−0.8564 (12)	0.069 (3)	
H11B	0.1206	−0.0042	−0.9113	0.104*	
H11A	0.0338	−0.0338	−0.8421	0.104*	
H11C	0.0968	−0.0999	−0.9032	0.104*	
C10	0.4033 (10)	0.2806 (6)	−0.2988 (13)	0.087 (5)	
H10B	0.3947	0.3149	−0.2238	0.130*	
H10A	0.3436	0.2455	−0.3299	0.130*	
H10C	0.4110	0.3157	−0.3733	0.130*	
O1W	0.5000	−0.2254 (4)	−0.2500	0.091 (5)	
H1W1	0.5281	−0.1936	−0.2981	0.137*	

### Atomic displacement parameters (Å^2^)

**Table:**

	*U*^11^	*U*^22^	*U*^33^	*U*^12^	*U*^13^	*U*^23^
Cu1	0.0194 (7)	0.0263 (6)	0.0300 (9)	0.000	−0.0015 (6)	0.000
O1	0.026 (3)	0.026 (2)	0.031 (4)	−0.0008 (18)	−0.012 (3)	−0.005 (2)
N1	0.033 (4)	0.032 (3)	0.030 (5)	−0.005 (2)	0.016 (4)	0.003 (3)
C1	0.018 (4)	0.038 (4)	0.030 (5)	0.001 (3)	0.003 (4)	−0.005 (3)
C2	0.031 (5)	0.037 (4)	0.049 (7)	0.000 (3)	−0.007 (5)	0.000 (4)
C3	0.020 (5)	0.060 (5)	0.052 (7)	−0.007 (3)	0.001 (5)	−0.014 (5)
C4	0.017 (4)	0.068 (5)	0.046 (7)	−0.005 (4)	−0.003 (5)	−0.003 (5)
C6	0.022 (4)	0.036 (3)	0.032 (5)	0.007 (3)	0.001 (4)	0.001 (3)
C7	0.030 (5)	0.029 (3)	0.036 (6)	0.007 (3)	0.006 (5)	0.011 (3)
C8	0.026 (5)	0.032 (4)	0.044 (6)	−0.007 (3)	−0.002 (5)	0.004 (3)
C9	0.046 (9)	0.025 (5)	0.073 (12)	0.000	0.018 (8)	0.000
C5	0.026 (5)	0.052 (4)	0.043 (6)	0.005 (3)	−0.002 (4)	0.007 (4)
C11	0.036 (6)	0.113 (8)	0.049 (8)	−0.013 (5)	−0.007 (6)	0.000 (6)
C10	0.123 (12)	0.058 (6)	0.089 (11)	0.050 (6)	0.045 (9)	0.034 (6)
O1W	0.106 (10)	0.033 (5)	0.106 (11)	0.000	−0.026 (8)	0.000

### Geometric parameters (Å, º)

**Table:**

Cu1—O1^i^	1.914 (4)	C6—C7	1.441 (10)
Cu1—O1	1.914 (4)	C7—H7	0.9300
Cu1—N1^i^	1.934 (6)	C8—C9	1.527 (10)
Cu1—N1	1.934 (6)	C8—H8A	0.9700
O1—C1	1.322 (9)	C8—H8B	0.9700
N1—C7	1.300 (10)	C9—C8^i^	1.527 (10)
N1—C8	1.480 (8)	C9—C10	1.533 (10)
C1—C6	1.403 (10)	C9—C10^i^	1.533 (10)
C1—C2	1.423 (10)	C5—H5	0.9300
C2—C3	1.377 (12)	C11—H11B	0.9600
C2—H2	0.9300	C11—H11A	0.9600
C3—C4	1.411 (13)	C11—H11C	0.9600
C3—H3	0.9300	C10—H10B	0.9600
C4—C5	1.382 (11)	C10—H10A	0.9600
C4—C11	1.507 (12)	C10—H10C	0.9600
C6—C5	1.413 (11)	O1W—H1W1	0.8513
			
O1^i^—Cu1—O1	91.0 (3)	N1—C8—C9	113.9 (7)
O1^i^—Cu1—N1^i^	93.9 (2)	N1—C8—H8A	108.8
O1—Cu1—N1^i^	155.2 (3)	C9—C8—H8A	108.8
O1^i^—Cu1—N1	155.1 (3)	N1—C8—H8B	108.8
O1—Cu1—N1	93.9 (2)	C9—C8—H8B	108.8
N1^i^—Cu1—N1	91.9 (4)	H8A—C8—H8B	107.7
C1—O1—Cu1	125.9 (4)	C8—C9—C8^i^	112.2 (8)
C7—N1—C8	118.8 (6)	C8—C9—C10	110.2 (6)
C7—N1—Cu1	125.8 (4)	C8^i^—C9—C10	106.9 (6)
C8—N1—Cu1	115.0 (5)	C8—C9—C10^i^	106.9 (6)
O1—C1—C6	124.5 (6)	C8^i^—C9—C10^i^	110.2 (6)
O1—C1—C2	117.8 (7)	C10—C9—C10^i^	110.5 (11)
C6—C1—C2	117.6 (8)	C4—C5—C6	123.4 (8)
C3—C2—C1	120.8 (8)	C4—C5—H5	118.3
C3—C2—H2	119.6	C6—C5—H5	118.3
C1—C2—H2	119.6	C4—C11—H11B	109.5
C2—C3—C4	122.5 (7)	C4—C11—H11A	109.5
C2—C3—H3	118.8	H11B—C11—H11A	109.5
C4—C3—H3	118.8	C4—C11—H11C	109.5
C5—C4—C3	115.8 (8)	H11B—C11—H11C	109.5
C5—C4—C11	121.0 (9)	H11A—C11—H11C	109.5
C3—C4—C11	123.1 (8)	C9—C10—H10B	109.5
C1—C6—C5	119.4 (7)	C9—C10—H10A	109.5
C1—C6—C7	123.4 (7)	H10B—C10—H10A	109.5
C5—C6—C7	117.1 (7)	C9—C10—H10C	109.5
N1—C7—C6	124.9 (7)	H10B—C10—H10C	109.5
N1—C7—H7	117.5	H10A—C10—H10C	109.5
C6—C7—H7	117.5		
			
O1^i^—Cu1—O1—C1	−166.5 (8)	C2—C1—C6—C5	−2.8 (12)
N1^i^—Cu1—O1—C1	92.1 (9)	O1—C1—C6—C7	−7.9 (13)
N1—Cu1—O1—C1	−10.9 (7)	C2—C1—C6—C7	176.4 (8)
O1^i^—Cu1—N1—C7	101.3 (8)	C8—N1—C7—C6	178.2 (8)
O1—Cu1—N1—C7	0.5 (8)	Cu1—N1—C7—C6	5.8 (13)
N1^i^—Cu1—N1—C7	−155.3 (9)	C1—C6—C7—N1	−3.6 (14)
O1^i^—Cu1—N1—C8	−71.3 (8)	C5—C6—C7—N1	175.7 (8)
O1—Cu1—N1—C8	−172.1 (6)	C7—N1—C8—C9	116.3 (8)
N1^i^—Cu1—N1—C8	32.1 (4)	Cu1—N1—C8—C9	−70.5 (7)
Cu1—O1—C1—C6	15.7 (11)	N1—C8—C9—C8^i^	35.3 (4)
Cu1—O1—C1—C2	−168.6 (6)	N1—C8—C9—C10	−83.7 (9)
O1—C1—C2—C3	−174.8 (8)	N1—C8—C9—C10^i^	156.2 (8)
C6—C1—C2—C3	1.2 (14)	C3—C4—C5—C6	6.1 (15)
C1—C2—C3—C4	4.2 (15)	C11—C4—C5—C6	−177.9 (9)
C2—C3—C4—C5	−7.7 (15)	C1—C6—C5—C4	−1.0 (14)
C2—C3—C4—C11	176.4 (10)	C7—C6—C5—C4	179.7 (9)
O1—C1—C6—C5	172.9 (8)		

Symmetry code: (i) −*x*+1, *y*, −*z*−1/2.

### Hydrogen-bond geometry (Å, º)

Cg1 is the centroid of the C1–C6 ring.

**Table:**

*D*—H···*A*	*D*—H	H···*A*	*D*···*A*	*D*—H···*A*
O1*W*—H1*W*1···O1	0.85	2.46	2.783 (7)	103
O1*W*—H1*W*1···O1^i^	0.85	2.44	2.783 (7)	105
C3—H3···O1*W*^ii^	0.93	2.55	3.48 (1)	173
C8—H8*B*···*Cg*1^iii^	0.97	2.83	3.693 (9)	148
C11—H11*B*···*Cg*1^iv^	0.96	2.98	3.850 (12)	151

Symmetry codes: (i) −*x*+1, *y*, −*z*−1/2; (ii) −*x*+1/2, −*y*−1/2, −*z*−1; (iii) *x*+3/2, *y*+1/2, *z*−1; (iv) −*x*+1/2, *y*+1/2, −*z*−1/2.
